# Congenital toxoplasmosis and auditory disorders: a literature review

**DOI:** 10.3389/fpsyg.2023.1286211

**Published:** 2024-01-04

**Authors:** Laís Ferreira, Milaine Dominici Sanfins, Julia Dalcin Pinto, Piotr Henryk Skarzynski, Magdalena B. Skarżyńska, Eliara Pinto Vieira Biaggio

**Affiliations:** ^1^Postgraduate Program in Human Communication Disorders, Department of Speech Therapy Federal University of Santa Maria, Santa Maria, Brazil; ^2^Department of Teleaudiology and Screening, Institute of Physiology and Pathology of Hearing, Kajetany, Poland; ^3^Albert Einstein Institute for Teaching and Research (IIEP), São Paulo, Brazil; ^4^Department of Pharmacotherapy and Pharmaceutical Care, Pharmaceutical Department, Medical University of Warsaw, Warsaw, Poland; ^5^ENT Department, Maria Curie-Skłodowska University, Lublin, Poland; ^6^Center of Hearing and Speech Medincus, Kajetany, Poland; ^7^Institute of Sensory Organs, Kajetany, Poland; ^8^Marie Curie-Skłodowska University, Lublin, Lublin Voivodeship, Poland; ^9^Center of Hearing and Speech, Nadarzyn, Poland

**Keywords:** hearing, electrophysiology, auditory evoked potentials, child development, congenital toxoplasmosis

## Abstract

**Background:**

Congenital toxoplasmosis (CT) occurs mainly by primary maternal infection during pregnancy. It is estimated that the incidence of vertical transmission to the fetus is 20% and that infected women are more likely to have a premature birth or low birth weight neonate since there is an association between CT and the rate of premature birth and low birth weight. In addition to severe neurological and ophthalmic consequences, hearing disorders such as hearing loss are also among the clinical manifestations seen in children with CT. Given the above, the objective of this study is to verify what are the auditory disorders seen in children with CT.

**Methods:**

This literature review was structured according to the PRISMA statement and based on the terms of Study Target Population, Intervention, Comparison, Outcomes, and Study Types (PICOS). To obtain the studies, the following electronic databases were consulted: PubMed, Web of Science, Scopus, and Lilacs. The combined terms used for the search were: (“auditory evoked potentials” OR “hearing” OR “hearing loss”) AND (“congenital toxoplasmosis”). The selection of articles was carried out independently, blindly, by two of the authors, to minimize risk of bias.

**Results:**

The search in the databases identified 172 articles, after excluding duplicate articles, 105 studies were identified. From the selection made by reading the titles and abstracts, 11 studies were selected for full-text reading. A total of 94 studies were excluded. An article was selected from the list of references. Therefore, 12 studies were included in the final analysis. It was observed that a significant percentage of studies sought to study the peripheral auditory pathway, verifying the occurrence or association between hearing loss and the presence of congenital infection. Only two studies evaluated the central auditory pathway, using the Brainstem Auditory Evoked Potential (BAEP) and the Frequency Following Response (FFR).

**Conclusion:**

Toxoplasmosis affects not only the peripheral areas but central areas as well. Most studies suggest this pathology as a risk factor for both peripheral and central impairment. Research has found a greater association between CT and mild to moderate hearing loss, in addition to alterations in exams such as BAEP and FFR. These data recommend that CT be reported as a global public health problem and can help assess complications and impacts of hearing disorders as a result of CT. There is a gap about studies that retract the co-occurrence between CT and other Risk Indicators for Hearing Loss (RIHL), such as prematurity, permanence in the intensive care unit, and use of ototoxic medications, lack of longitudinal studies, that accompany the development of hearing and language of children with CT, since the consequences of this infection may be late.

## 1 Introduction

Congenital toxoplasmosis (CT) occurs mainly by primary maternal infection during pregnancy and brings an economic and health burden for affecting many pregnant women, especially those with low and middle income in underdeveloped countries. It is estimated that the incidence of vertical transmission to the fetus is 20% (Li et al., 2014) and that infected women are more likely to have a premature birth or the low birth weight neonate, since there is an association between CT and the rate of premature birth and low birth weight (Hurt et al., 2022). In addition, congenital and perinatal infections affect 0.5 to 2.5% of neonates and are the main causes of infant morbidity and mortality, complications of premature birth are the main cause of death in children under 5 years old (Liu et al., 2016).

The clinical diagnosis in this period is a challenge for professionals in the area since most cases are asymptomatic. However, the consequences are significant for the health of the child population and may occur in the neonatal period or late sequelae (Mirambo et al., 2019).

The clinical manifestations observed by this pathology depend on factors such as the mode of transmission, time of infection in relation to gestational age, maturity of the fetal immune system, and presence or absence of maternal immunity at the time of infection (Menson and Lyall, 2005).

In addition to severe neurological and ophthalmic consequences, hearing disorders are also among the clinical manifestations observed in children with CT (Al-Amari and Kameswaran, 1996; Andrade et al., 2008; de Resende et al., 2010; Vos et al., 2015; Besen et al., 2022). When analyzing the histopathology of the temporal bone in children with CT, researchers described that hearing loss in this infection may be a consequence of post-natal inflammation, since they found an inflammatory process, triggered by the parasite, in the inner ear (Salviz et al., 2013).

Although recommendations for children’s hearing health (Lewis et al., 2010; Joint Committee on Infant Hearing, 2019) cite CT and prematurity as Risk Indicators for Hearing Loss (RIHL), the results found in studies are variable. Thus, there is no consensus in the literature between the presence of hearing loss and this infection. While some authors report that such infection is a RIHL in children (Al-Amari and Kameswaran, 1996; Andrade et al., 2008; de Resende et al., 2010; Vos et al., 2015; Besen et al., 2022), other studies do not find this association (Lipka et al., 2002; Austeng et al., 2010).

Given the above, the objective of this study is to verify what are the auditory disorders observed in children with CT.

## 2 Materials and method

This literature review was structured according to the Preferred Reporting Items for Systematic Reviews and Meta-Analyses (PRISMA) statement and based on the terms of PICOS (Participants, Interventions, Comparators, Outcomes, and Study Design).

### 2.1 Eligibility criteria

Studies based on PICOS terms that evaluated the association between congenital toxoplasmosis and the presence of auditory disorders were included. PICOS stands for Study Target Population, Intervention, Comparison, Outcomes, and Study Types. Target population (P) corresponds to children; intervention (I) relates to congenital infections; comparison (C) was considered as children without congenital infections and typical audiological development; outcome (O) refers to the existence or not of auditory disorders; and the types of studies admitted (S) were observational, cohort, cross-sectional and randomized clinical trials. No restrictions were applied regarding language and year of publication. The studies should also obtain a score greater than 80% in the Study Quality Assessment Tools, showing that this is a high-quality study.

Studies that did not address the subject of this review, which were not carried out with the target population, reviews, letters, books, conference abstracts, case reports, case series, systematic and literature reviews, and opinion articles were excluded.

### 2.2 Search strategy

To obtain the studies, the following electronic databases were consulted: PubMed, Web of Science, Scopus and Lilacs. The research was carried out between May 2023 and June 2023, with individual search strategies by two researchers. Manual searches were also performed in bibliographic references in order to include relevant studies.

The combined terms used for the search were: (“auditory evoked potentials” OR “hearing” OR “hearing loss”) AND (“congenital toxoplasmosis”). Filters referring to the year of publication and language were not applied, however, filters related to the age of the sample were applied.

### 2.3 Selection of studies

The selection of articles was carried out independently, blindly, by two of the authors, avoiding any risk of bias. In this way, the names of authors and journals were masked to avoid any potential bias and conflict of interest. The articles were, at first, selected by title and abstract and were excluded for not respecting at least one of the inclusion criteria. The remaining articles were read in full and evaluated for eligibility. Any disagreement was resolved by discussion.

### 2.4 Risk of bias in individual studies

The risk of bias analysis was also performed independently, blindly, by two of the authors and subsequently agreed between them and a third author. For the analysis, the questionnaires of the Systematic reviews of etiology and risk instruments were used, specifically the Critical appraisal checklist for analytical cross-sectional studies and the Critical appraisal checklist for cohort studies. The final quality of each article was categorized by the following scores of answers “Yes,” “No” and “Little applicable,” being >70%, low risk of bias; between 50 and 69%, moderate risk of bias; <50%, high risk of bias.

### 2.5 Data extraction, synthesis and analysis

The information extracted from the studies were: authors, year of publication, age group studied, sample size, objective, results, and conclusion (Table 1) to then evaluate the association between congenital infections and hearing disorders. The extracted data were analyzed descriptively and comparatively. When any of this information was not available, the corresponding author was contacted by email.

**TABLE 1:** Description of the included studies, presented in descending order of publication.

Author/year of publication	Sample/Procedure	Objective	Results/Conclusion
Ferreira et al., 2020	A total of 11 infants diagnosed with CT and 12 healthy infants, of both sexes, aged between 29 and 90 days of life; FFR.	To investigate the effect of CT on the FFR electrophysiological response in infants.	Infants with CT showed increased latency of V, A, E, F, and O waves, and decreased amplitude of A and F waves. They also showed a reduction in AO slope and a greater difference in latency between onset (A) and displacement (O).
Fontes et al., 2019	A total of 76 children, age range of 1–3 months of age; 37 diagnosed with CT; BAEP.	To evaluate and describe BAEP in babies aged one to three months diagnosed with CT and compare with babies of the same age group without the infection.	Two children without toxoplasmosis and 10 with CT presented results suggestive of alteration in the maturational process of the brainstem auditory pathway. Thus, 10 (27%) children were identified with possible unilateral alteration in the electrophysiological assessment and a five times greater risk of a child between one and three months old with toxoplasmosis presenting alteration in BAEP when compared to a child of the same age group without the infection.
Noorbakhsh et al., 2019	A total of 45 cases with the cochlear implant surgery (Idiopathic profound SNHL) and 30 controls; Blood samples.	To determine immunity to *T. gondii* and CMV in children with cochlear implants.	*T. gondii*-IgM positive was found within 8/45 (17.7%) and also one of these cases (2.2%) had *T. gondii*–IgG Test. *T. gondii* was observed to have a relative role within younger cases with profound SNHL, but a greater role within mild to moderate SNHL.
Al Sabellha and Hager, 2012	A total of 168 patients with profound SNHL; Review of TORCH laboratory.	To verify the effectiveness of the TORCH investigation (toxoplasma, rubella, cytomegalovirus, herpes simplex) in children with profound SNHL.	Of the 168 patients, 102 had TORCH laboratory results. Although, no patient tested positive for toxoplasma or syphilis.
Austeng et al., 2010	A total of 27,727 children born in Norway between the years 1992–1994; Pre- and post-natal serological examination.	To investigate the association between maternal *T. gondii* infection during pregnancy and subsequent risk of AP in the offspring.	22 of the 27,727 children (0.08%) had HL. Forty women had *T. gondii* in pregnancy and none of their offspring had PA. There was also no association between *T. gondii* infection before pregnancy and AP in the offspring.
de Resende et al., 2010	A total of 146,307 newborns; Behavioral observation audiometry, BAEP, transient and distortion product otoacoustic emission and tympanometry.	To describe the audiological and language evolution in children with diagnosed CT and treated early.	106 children were diagnosed with CT, of which 60 had normal hearing (56.6%) and 46 had altered hearing, 13 (12.3%) with conductive alteration, 4 (3.8%) with SNHL and 29 (27, 4%) with retrocochlear involvement. There was an association between the presence of hearing alteration and language deficit. Even with early diagnosis and treatment, a high prevalence of audiological impairment was observed in children with CT.
Andrade et al., 2008	A total of 20 children identified with CT; Behavioral audiometry, evoked otoacoustic emission and BAEP.	Assess the hearing of children with CT identified by neonatal screening.	20 in 30.808 children presented CT, of which 15 (75%) had subclinical infection. SNHL was present in four children (21.1%), of which only one had more than one RIHL and three presented only the CT as RIHL. Two children, properly treated with antiparasitics, presented hearing deficits as well.
Lipka et al., 2002	A total of 38 children diagnosed with CT and 34 diagnosed with CMV, with visual/auditory impairment between the years 1995–2001; Medical record analysis.	To assess the character and frequency of auditory and visual disturbances in children with CT and CMV infection.	Visual impairment was observed in all children with toxoplasmosis (with a visual impairment rate of 74%), but deafness was not found. The rate of neurological deficits was much higher in children with toxoplasmosis (52% vs. 4%).
de Azevedo et al., 2000	A total of 24 children, aged between 0 to 2 years; Behavioral audiometry and CAP assessment.	To verify the prevalence of peripheral HL and/or CAP alteration in children from 0 to 2 years of age, with CT.	54.2% of the children did not present auditory alteration, 12.5% presented transitory peripheral alteration and 33.3% CAP alteration. In view of the findings, it can be concluded that CT is an important etiological factor for CAP alteration.
Al-Amari and Kameswaran, 1996	A total of 50 congenitally deaf Saudi males between the ages of 11 and 30 months (GROUP A). A group of 50 Saudi males with normal hearing (GROUP B); Serological tests.	To assess the incidence of positive serology for toxoplasma in a group of congenitally deaf children in Asir Province, southern Saudi Arabia.	There seems to be strong circumstantial evidence to consider CT as one of the important factors in the etiology of congenital deafness in our population.
McGee et al., 1992	A total of 30 congenitally infected infants and children; BAEP.	To verify the occurrence of hearing loss in children with treated CT.	The preliminary results we describe suggest that HL may be less frequent than previously reported in children with treated CT. This could be because of the prolonged antimicrobial therapy these children received.
Stagno et al., 1977	A total of 38 children with CT; Medical record analysis.	To verify the incidence of auditory and visual deficits resulting from congenital infections by cytomegalovirus and toxoplasma	HL was present in ten of 59 patients with CMV infection and in 5 of 12 patients with CT. These data establish congenital infections as an important public health problem and confirm the fact that CT may be associated with late-appearing deficits.

BAEP, Brainstem Auditory Evoked Potential; CT, Congenital toxoplasmosis; SNHL, sensorineural hearing loss; RIHL, Risk indicator for hearing loss; CAP, Central Auditory Processing; HL, Hearing loss; *T. gondii, Toxoplasma gondii;* CMV, cytomegalovirus.

## 3 Results

### 3.1 Selected studies

In the blind selection of articles, the researchers identified 172 articles. After removing duplicates, 105 studies were selected for eligibility according to the criteria previously established. Finally, 11 studies were selected for full-text reading after careful title and abstract screening. A total of 94 studies were excluded (16 for being a systematic review, eight for being case studies, one for not finding the full text and 70 for not addressing the proposed topic). An article was selected from the list of references. Therefore, 12 studies were included for the final analysis. Supplementary Figure 1 demonstrates the study selection process in detail.

### 3.2 Characteristics of the studies

The selected studies were published between 1977 and 2021, with a greater number of publications between 2000 and 2010. All studies were published in English, four of which are also available in Portuguese. As for the design of the studies, 13 cross-sectional studies and one cohort study were selected. The vast majority of studies evaluated children from 1 day of life to 2 years of age.

It was observed that a significant percentage of studies sought to study the peripheral auditory pathway, verifying the occurrence or association between hearing loss and the presence of congenital infection. Only two studies evaluated the central auditory pathway, using the Brainstem Auditory Evoked Potential (BAEP) and the Frequency Following Response (FFR).

The authors verified the existence of both hearing loss (Al-Amari and Kameswaran, 1996; de Azevedo et al., 2000; Andrade et al., 2008; de Resende et al., 2010) and increased latency values in the BAEP and FFR responses (de Resende et al., 2010; Fontes et al., 2019; Ferreira et al., 2020). Therefore, the auditory pathway of children with CT can be affected both at the peripheral and central hearing levels.

To illustrate the analyzed studies, a table was prepared with the description of each one of them, with the identification of authorship, year of publication, title, objective, results and conclusion (Table 1).

### 3.3 Quality assessment

The quality assessment of the studies indicated that most of the studies were considered at low risk of bias, two studies were categorized with moderate risk of bias and one with a high risk of bias. Table 2 shows the details of this analysis.

**TABLE 2:** Risk of bias analysis based on the instrument’s questionnaires.

Cross-sectional studies	Inclusion criteria	Description of subjects and the study	Exposure Measurement	condition measurement	Confounding factors	Strategies of confounding factors	Results	Statistical analysis
Ferreira et al., 2020	Yea	Yea	Yea	Yea	No	Not applicable	Yea	Yea
Fontes et al., 2019	Yea	Yea	Yea	Yea	No	Not applicable	Yea	Yea
Noorbakhsh et al., 2019	Yea	Yea	Yea	Yea	No	Not applicable	Yea	Yea
Al Sabellha and Hager, 2012	unclear	unclear	unclear	unclear	No	Not applicable	unclear	unclear
Austeng et al., 2010	Yea	Yea	Yea	Yea	No	Not applicable	Yea	Yea
de Resende et al., 2010	Yea	unclear	Yea	Yea	No	Not applicable	Yea	Yea
Andrade et al., 2008	Yea	Yea	Yea	Yea	No	Not applicable	Yea	Yea
Lipka et al., 2002	Yea	unclear	Yea	Yea	No	Not applicable	Yea	Yea
de Azevedo et al., 2000	Yea	Yea	Yea	Yea	No	Not applicable	Yea	Yea
Al-Amari and Kameswaran, 1996	Yea	Yea	Yea	Yea	No	Not applicable	Yea	Yea
McGee et al., 1992	unclear	unclear	Yea	Yea	No	Not applicable	Yea	Yea
Stagno et al., 1977	unclear	unclear	Yea	Yea	No	Not applicable	Yea	Yea

## 4 Discussion

Together, the data found in this review show that children with congenital toxoplasmosis may have both hearing loss (Al-Amari and Kameswaran, 1996; de Azevedo et al., 2000; Andrade et al., 2008; de Resende et al., 2010) and alterations in central auditory pathways (de Resende et al., 2010; Fontes et al., 2019; Ferreira et al., 2020). Thus, this infection should be pointed out as a risk for disturbances in the peripheral and central auditory pathway. This finding suggests children with CT should undergo, besides typical audiological assessments, other more specific procedures for differential diagnosis.

Regarding the presence of hearing loss in children with CT, the literature does not demonstrate a consensus, as previously mentioned. While some authors found an association with hearing loss (Al-Amari and Kameswaran, 1996; de Azevedo et al., 2000; Andrade et al., 2008; de Resende et al., 2010) and language disorders (de Resende et al., 2010), another study did not find any association (Austeng et al., 2010).

Still regarding the degree of sensory auditory deficit, there is no significant relationship that children with congenital toxoplasmosis have profound hearing loss (Noorbakhsh et al., 2019). There is a greater correlation between children with CT and cases of mild to moderate hearing loss (Noorbakhsh et al., 2019).

Similar data were found in two studies that sought to understand the relationship between CT, hearing loss and possible changes in the Central Auditory Processing (CAP) (de Azevedo et al., 2000; de Resende et al., 2010). The results of these studies indicated that 54.2% to 56.6% of children with CT do not have auditory alterations, 12.3% to 12.5% have transient and conductive alterations, 3.8% have peripheral sensorineural alterations, 27.4% have retrocochlear changes and 33.3% have CAP changes (de Azevedo et al., 2000; de Resende et al., 2010).

In view of this, it is inferred that children with CT may demonstrate alterations in the basic diagnosis of hearing, evidenced by both conductive and sensorineural hearing loss. When performing differential assessments, children may also manifest changes in CAP.

The presence of clinical neurological manifestations in children with CT makes it essential to carry out the differential diagnosis of hearing. Through the evaluation of the central auditory nervous system, research has shown that children with CT may present alterations both at the brainstem level and at the subcortical and cortical levels (de Resende et al., 2010; Fontes et al., 2019; Ferreira et al., 2020).

At the brainstem level, the literature highlights that a child with CT, aged between 1 and 3 months, has a five times greater risk of manifesting an alteration in BAEP when compared to a child without the disease (Fontes et al., 2019). Furthermore, alterations at the subcortical and cortical level were verified through the FFR evaluation. The authors report that the group of children with CT showed a statistically significant increase in the latency of V, A, E, F, and O and a decrease in the amplitude of the A and F waves (Ferreira et al., 2020). Another interesting fact, when evaluating a group of children only exposed to toxoplasmosis during pregnancy, the authors observed that these children did not differ from the non-exposed group in terms of cochlear and conductive hearing loss, although they presented a higher occurrence of possible retrocochlear alteration (Leite Filho et al., 2017).

Researchers, who report neurophysiological delays in the central auditory pathway of children with CT when compared to neurotypical children, do not detail whether the group with the infection is behavior for term-born children. Considering that prematurity can cause immaturity in the auditory pathway and consequently delay in the latency of the waves of such potentials, this discussion would be interesting.

Given these data, the relevance of evaluations classified as differential diagnosis in this population is highlighted. It can be concluded that toxoplasmosis does not affect only the peripheral portion of the auditory pathway and that other evaluations deserve attention. However, more research is needed to establish in more detail the links and reflexes of congenital toxoplasmosis in the central auditory nervous system.

A limitation observed among the studies in this literature review is the restriction in relation to birth and health data and the demographic profile of the participants, which made it difficult to detail the characteristics of the population. There is a lack of research that portrays the co-occurrence between CT and other RIHLs, such as prematurity, permanence in the intensive care unit and use of ototoxic medications, in addition to reporting whether the existence of another RIHL increases or not the chances of hearing disorders.

In addition, another gap is the lack of longitudinal studies that follow the development of hearing and language of children with CT, since the consequences of this infection may be late. Therefore, it is recommended that studies be conducted to provide information regarding central auditory nervous system evaluations and to correlate the auditory disorders present in this population with their impact on the language of these children.

However, understanding the importance of an integral peripheral and central auditory system for the child to have an adequate acquisition and appropriation of language, such findings lead to a concern regarding child development. For this reason, the importance of audiological and language monitoring within newborn hearing screening programs is highlighted, as already mentioned in the literature (Lewis et al., 2010; Joint Committee on Infant Hearing, 2019).

## 5 Conclusion

Given these data, the relevance of evaluations classified as differential diagnosis in this population is highlighted. It can be concluded that toxoplasmosis does not affect only the peripheral and most studies suggest that this pathology is a risk factor for peripheral and central impairment. Research has found a greater association between CT and mild to moderate hearing loss, in addition to alterations in exams such as BAEP and FFR. These data recommend that CT be reported as a global public health problem and can help assess complications and impacts of hearing disorders as a result of CT. In addition to serving as support for the development of guidelines for child health services.

## Data availability statement

The original contributions presented in this study are included in this article/Supplementary material, further inquiries can be directed to the corresponding author.

## Author contributions

LF: Conceptualization, Data curation, Investigation, Methodology, Project administration, Writing−original draft, Writing−review and editing. MDS: Formal analysis, Supervision, Writing−review and editing. JP: Data curation, Investigation, Methodology, Writing−original draft, Writing−review and editing. PS: Funding acquisition, Methodology, Resources, Writing−review and editing. MS: Funding acquisition, Methodology, Resources, Writing−review and editing. EV: Conceptualization, Data curation, Formal analysis, Investigation, Methodology, Project administration, Supervision, Writing−review and editing.
