# Supplementary material for: Congenital toxoplasmosis and auditory disorders: a literature review
Source: Front Psychol. 2024 Jan 4;14:1286211. doi: 10.3389/fpsyg.2023.1286211 (PMC10828674; doi:10.3389/fpsyg.2023.1286211)

Supplementary Material

Supplementary. Figure 1 – Identification process of studies for inclusion in the literature review.

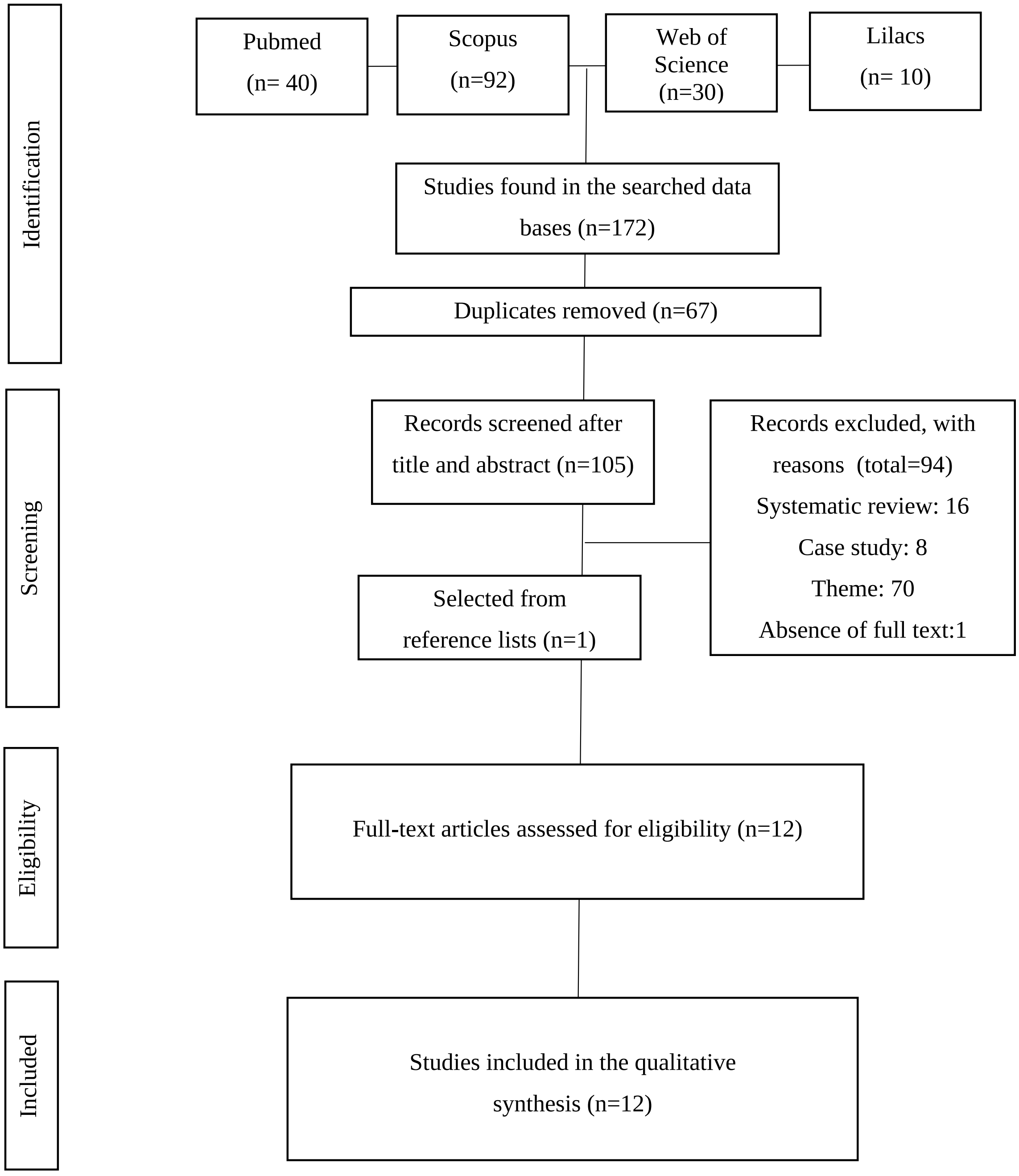

Supplement: Supplementary file 1 [file Image_1.pdf]
